# Risk Factors for Lymphatic Metastasis of Malignant Bone and Soft-Tissue Tumors: A Retrospective Cohort Study of 242 Patients

**DOI:** 10.1097/MD.0000000000000225

**Published:** 2014-12-12

**Authors:** Takashi Yanagawa, Kenichi Saito, Kenji Takagishi

**Affiliations:** From the Department of Orthopaedic Surgery, Gunma University Graduate School of Medicine, Showa, Maebashi, Gunma, Japan.

## Abstract

Metastasis to the lymph nodes is relatively rare in malignant bone and soft-tissue tumors, and its risk factors remains unknown, except for tumors of the lymphogenous histotype, including rhabdomyosarcoma, epithelioid sarcoma, and clear cell sarcoma. The purpose of this study was to identify the risk factors for lymph node metastasis of malignant bone and soft-tissue tumors.

We retrospectively reviewed 242 patients with malignant bone and soft-tissue tumors. The predictors of interest for the risk of lymph node metastasis included age, sex, histopathological diagnosis, location(s) of the primary tumor(s), local recurrence, residual tumor(s), and the size of the primary tumors. To identify the risk factors for lymph node metastasis, Cox regression analyses were performed.

Among the 242 patients with malignant bone and soft-tissue tumors in the current study, 60, 29, and 28 were detected to have lung, lymph node, and bone metastases, respectively. In the univariate analyses, the lymphogenous histotype and a primary tumor invading the subcutis were the risk factors for lymph node metastasis. In the multivariate analysis, the lymphogenous histotype (*P* < 0.01) and a primary tumor in the subcutis (*P* < 0.01) remained significantly associated with a higher risk of lymph node metastasis with 5.15 and 3.48 of hazard ratios, respectively.

Lymph node metastasis was detected in malignant bone and soft-tissue tumors more frequently than that has been previously reported, and the risk factors for lymph node metastasis were the lymphogenous histotype and primary tumors invading the subcutis.

## INTRODUCTION

Approximately, 14,000 malignant bone and soft tissue tumors are newly diagnosed each year in the United States, and about 5000 patients die of the disease each year.^1^ The 5-year survival rate of patients with osteosarcomas without metastasis is about 70%,^2,3^ while that of patients with metastasis is about 30%.^4^ Malignant soft-tissue tumors are reported to be associated with 5-year survival rates of about 80% and 10% for patients without and with metastasis, respectively.^5,6^ Therefore, controlling the metastasis is essential to successfully treat malignant bone and soft-tissue tumors.

Previous studies described that the risk factors for the distant metastasis of malignant soft-tissue tumors were the tumor grade, tumor size, tumor depth, and local recurrence, and most of the reports focused on lung metastasis because of its high frequency.^6–9^ Lymph node metastasis is a relatively rare event in malignant bone and soft-tissue tumors, but there are some exceptions, such as rhabdomyosarcoma, epithelioid sarcoma, and clear cell sarcoma.^6,10–12^ Zagars et al defined these 3 histotypes as “lymphogenous histotypes,” and this classification is an independent risk factor for lymph node metastasis of malignant soft-tissue tumors.^6^ Until now, no clinical factors other than the histotype have been reported as predictors of lymph node metastasis in patients with malignant bone and soft-tissue tumors.

Some previous reports have suggested that the lymphangiogenesis elicited by factors produced by tumors was relevant to tumor cell invasion of the lymphatic vasculature and subsequent metastasis.^13,14^ Vascular endothelial growth factor (VEGF)-C and -D enhance lymphangiogenesis via their receptor, VEGF receptor (VEGFR)-2, and -3.^15–17^ Recently, these proteins were found to disrupt the barrier function of the lymphatic endothelium and to augment the transendothelial migration of sarcoma cells in vitro.^18^ This suggested that targeting VEGF-C, -D, VEGFR-2, and -3 might prevent the development of lymphatic metastasis in patients with malignant bone and soft-tissue tumors.^19,20^ However, lymphatic metastasis is a relatively rare event in patients with malignant bone and soft-tissue tumors, and therefore, targeted therapy should be applied only for high-risk patients, rather than to all patients, following an evaluation of the risk factors for lymphatic metastasis.

The purpose of this study was to identify the risk factors for lymphatic metastasis using clinical data and to contribute to treatment for malignant bone and soft-tissue tumors.

## METHODS

### Patients and Treatment

After obtaining Institutional Review Board approval, we retrospectively reviewed 290 patients with bone and soft-tissue sarcomas in their extremities and trunk, except the spinal cord, who visited our hospital from 2003 to 2012. We excluded 48 patients because their tumors were diagnosed as intermediate grade, such as well-differentiated liposarcoma and studied the remaining 242 patients.

To screen for metastasis, all patients were examined by chest X-rays, CT, MRI, and/or 2-deoxy-2-[F-18]fluoro-d-glucose positron emission tomography (FDG-PET) prior to treatment. Whole body FDG-PET or CT was performed at 6-month to 1-year intervals for 3 years after treatment. We defined lymph node metastasis as the appearance of a high FDG accumulation (standardized uptake value > 4.5) in lymph nodes in FDG-PET according to previous reports, and as growing lymph nodes on CT.^21,22^ All images were independently interpreted by 2 experienced diagnostic radiologists. Pathological diagnoses were also obtained for the cases in which lymph nodes were resected. Patients with soft-tissue sarcoma who had no metastasis received no chemotherapy, however, patients with metastasis received treatment with doxorubicin or pirarubicin at 30 to 60 mg/m^2^ and ifosfamide at 10 to12 g/m^2^ after providing informed consent. Patients with osteosarcoma and Ewing's sarcoma received pre- and postoperative chemotherapy according to NECO95J protocol and with VDC/IE (a regimen of alternating vincristine–doxorubicin–cyclophosphamide and ifosfamide–etoposide), respectively.^3,23^ Patients with a resectable tumor underwent surgery, and the surgical margin was histologically evaluated. When the tumors were inoperable or the surgical margins were not sufficient, radiation therapy, including carbon ion radiotherapy, was given. The radiotherapy dose ranged from 50 Gy/25 to 66 Gy/33 fractions and palliative radiation was administered at 30 Gy in 10 fractions. The carbon ion dose was 64.0 to 70.4 Gy (relative biological effectiveness) given in a total of 16 fixed fractions. After treatment, patients were followed at 3- to 6-month intervals for the first 5 years and yearly thereafter. Palliative treatment was administered to patients whose general condition was too poor for chemotherapy, radiotherapy, or surgery.

Informed consent was obtained from all patients. In our institute, most of the patients signed a comprehensive consent form stipulating that their information related to treatment may be used for research and published, without revealing their identity, at the first visit. We developed another consent form specific to the current study and obtain the consent from the patients or their family that allowed us to use their information, so the comprehensive consent form was only used for the patients who were difficult to contact. This study was approved by the Institutional Review Board in Gunma University Hospital on January 23, 2013, and was assigned registration number 1005.

### Predictors of Interest

**FIGURE 1:**
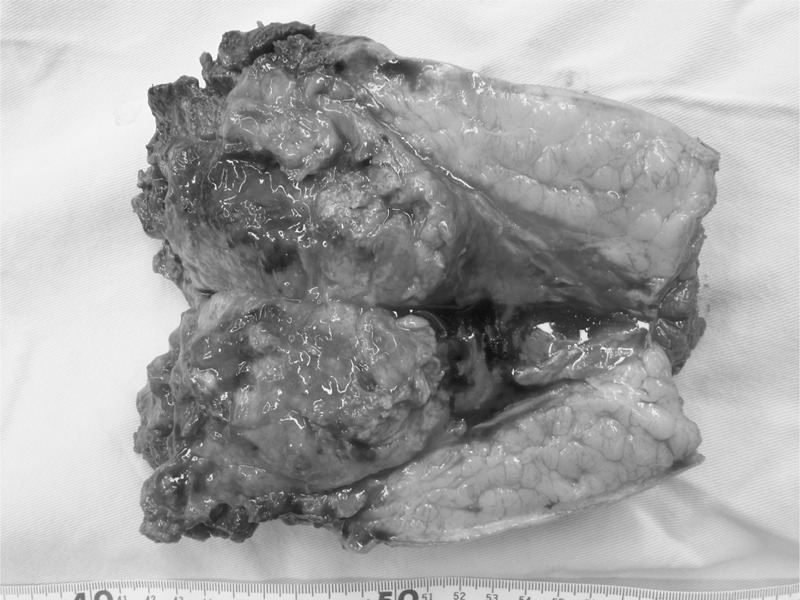
A rhabdomyosarcoma in a gluteus maximus muscle that invaded the subcutis. The regions that the tumor involved were the muscle and subcutis.

The predictors of interest for the risk of lymph node metastasis included age, sex, histopathological diagnosis, location(s) of the primary tumor, local recurrence, residual tumor, and the size of the primary tumor. According to the report by Zagars et al,^6^ the age was categorized as ≤64 or >64. Zagars et al previously reported that rhabdomyosarcoma, epithelioid sarcoma and clear cell sarcoma more frequently metastasized to lymph nodes than other sarcomas, and classified these cancers as having lymphogenous histotypes.^6^ In the current study, the histopathological diagnosis was categorized as either the lymphogenous histotype or others. In addition, for the histopathological tumor types of which there were more than 15 cases; undifferentiated pleomorphic sarcoma, liposarcoma, osteosarcoma, chondrosarcoma, and myxofibrosarcoma, we also evaluated the histopathological type as a predictor of interest. The locations where the primary tumors invaded were categorized into 3 groups; the subcutis, muscle, and bone. Some tumors were present in 3 or 3 locations or regions. For example, when a primary tumor in muscle was infiltrated into the subcutis, the location of the tumor was defined as the muscle and subcutis (Figure 1). The locations involved by the tumors were judged from the histopathological findings of the resected tumors for the patients who underwent surgery and from the radiological findings for the patients who had unresectable tumors. Tumors with a microscopically positive margin and tumors without surgery or radiotherapy were defined as residual tumors. The primary tumor size was recorded as the greatest tumor dimension before treatment. According to the American Joint Committee on Cancer criterion, the tumor size was categorized as ≤5 cm (T1) or >5 cm (T2) and ≤8 cm (T1) or >8 cm (T2) for soft-tissue and bone tumors, respectively.

### Statistical Analyses

We calculated that we would need a sample size of at least 196 patients to detect a 10% difference (85–95%)^10,11^ in the 3-year lymph node metastasis-free survival at a 5% type-I error rate and with 80% power for a two-tailed log-rank test in which the ratio of sample sizes in the 2 groups was 3. Kaplan–Meier curves were generated for the time to lymph node metastasis, and the risk of metastasis was analyzed univariately by log-rank tests. Only variables that were associated with metastasis at a level of *P* < 0.05 were considered for entry into a Cox proportional hazards model.

Calculation of the sample size was performed with EZR (Saitama Medical Center, Jichi Medical University, Saitama, Japan),^24^ which is a graphical user interface for the R software program (The R Foundation for Statistical Computing, Vienna, Austria), and other statistical analyses were performed using the SPSS software program (version 22.0; IBM Corporation, Somers, NY).

## RESULTS

### Patient and Tumor Characteristics

There were 147 males and 95 females with a mean age of 57.0 ± 20.7 (SD) years and a mean follow-up of 32.4 ± 27.0 (SD) months included in our study. The histopathological diagnoses are shown in Table 1. Thirteen patients had a sarcoma defined as lymphogenous histotype, including rhabdomyosarcoma, epithelioid sarcoma, and clear cell sarcoma. Twenty-seven of the patients received no surgical treatment other than biopsy. The pathological or radiological findings showed that 155, 86, and 83 primary tumors were located in the muscle, subcutis, and bone, respectively. Among them, 76 and 4 tumors invaded 2 and 3 of the regions, respectively. There were 120 and 54 cases in which the tumor was categorized as T2 in soft-tissue tumors and bone tumors, respectively. The data on the size and location of the primary tumors were missing for 2 patients because they underwent surgery at other hospitals, and the descriptions of the tumors were insufficient.

**TABLE 1:**
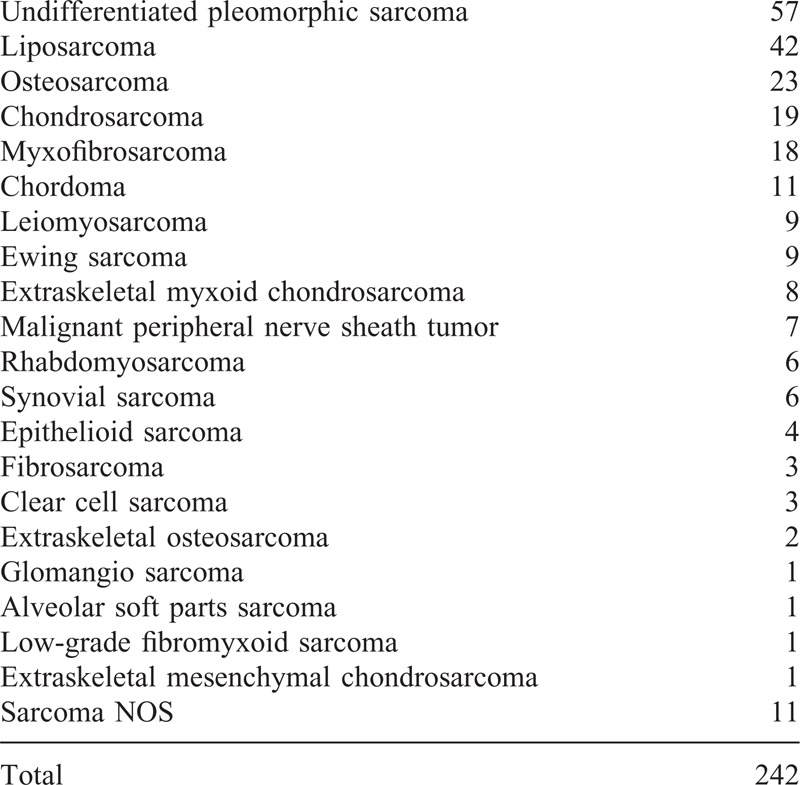
The Pathological Diagnosis of the Tumors

### Treatment

The surgical margins were microscopically evaluated, and 198 were diagnosed to be negative and 17 were considered to be positive. Among the patients who underwent surgery 8 patients underwent surgery before visiting our hospital. Radiotherapy without surgery was performed for 17 patients. The remaining 10 patients received palliative treatment without surgery or radiotherapy because of their poor general condition. Consequently, 27 patients had tumors with a positive margin or tumors without treatment, which were defined as residual tumors.

### The Frequency of Metastasis and Local Recurrence

Of the 242 patients with bone and soft-tissue sarcoma evaluated in the current study, 60, 29, and 28 patients had lung, lymph node, and bone metastases, respectively. Among them, 4 patients had metastases to the lung, lymph nodes, and bones, and 10, 5, and 5 patients had lung and lymph node metastases, lung and bone metastasis, and lymph node and bone metastases, respectively. Local recurrence developed in 57 cases after surgery, radiotherapy or both, and 11 patients subsequently experienced lymph node metastasis.

### Risk Factors for and Predictors of Lymph Node Metastasis

In the univariate analyses, the lymphogenous histotype and a primary tumor invading the subcutis were significantly associated (*P* < 0.05) with a higher hazard ratio for lymph node metastasis. In the multivariate analysis that included the variables with a value of *P* < 0.05 in the univariate analysis, the lymphogenous histotype and a primary tumor invading the subcutis remained significantly associated with a higher risk of lymph node metastasis, and the hazard ratios were 5.15 with a 95% confidential interval (CI) ranging from 2.27 to 11.67 (*P* < 0.01) and 3.48 (95% CI = 1.62–7.49, *P* < 0.01), respectively (Table 2).

**TABLE 2:**
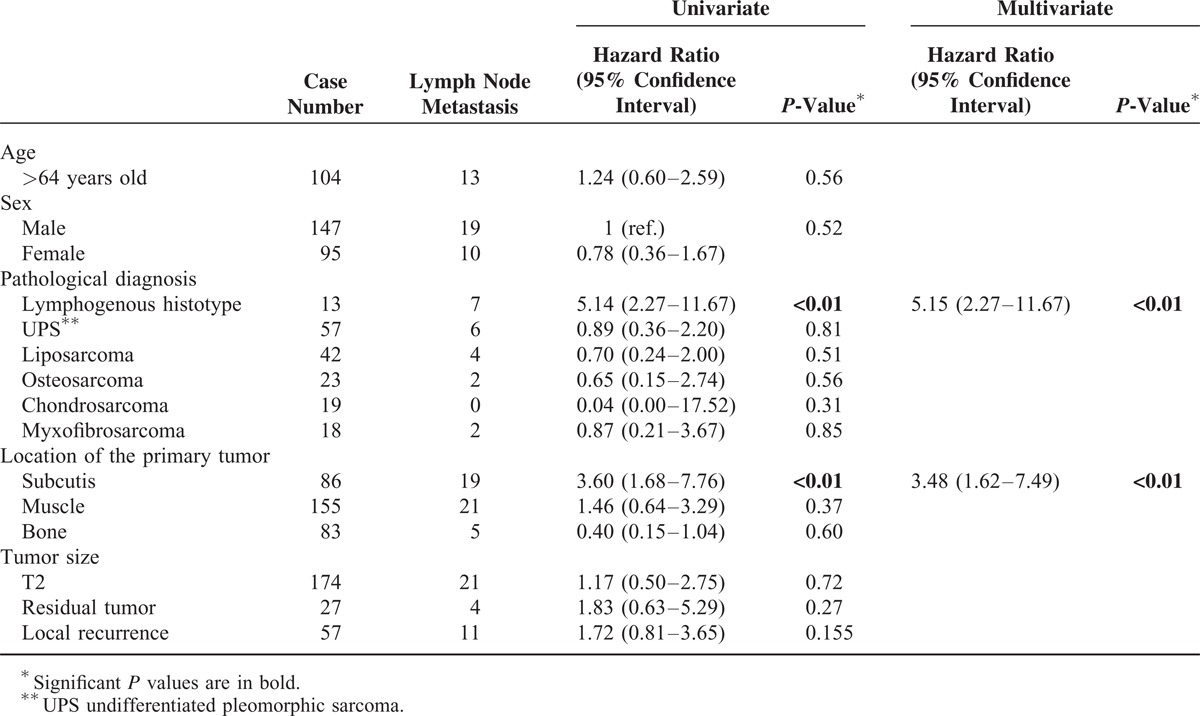
Factors Associated With Lymph Node Metastasis of Malignant Bone and Soft-Tissue Tumors

## DISCUSSION

The current study revealed that the risk factors for lymph node metastasis in patients with malignant bone and soft-tissue tumors were lymphogenous histotypes and a primary tumor invading the subcutis based on the multivariate Cox regression analysis. The histological location of the primary tumor had not previously been considered a risk factor for metastasis. Of note, our analysis elucidated that malignant bone and soft-tissue tumors involving a subcutaneous region had a propensity to metastasize to the lymph nodes. Lachenmayer et al^25^ reported that superficial soft-tissue sarcomas located above the muscle fascia metastasized to the lungs most frequently and rarely to lymph nodes; however, they excluded patients whose tumor had invaded the muscle fascia and patients who had metastasis before treatment. In this study, 46 of 86 cases involving the subcutis were located in only the subcutaneous region, while the remaining 40 cases consisted of subcutaneous tumors invading muscles and bones and intramuscular tumors invading the subcutis. Lymph node metastases were observed in 7 of the 46 cases without invasion (15.2%) and in 12 of the 40 cases with invasion (30%), respectively, suggesting that tumor cells that invaded the deep fascia probably were more aggressive and metastasized easily.

The skin and mucous membranes of the gastrointestinal and respiratory tracts, which are frequently in contact with foreign antigens, are particularly rich in lymphatic vessels.^20^ The lymphatics are localized to the fascia plane and do not enter the muscle bundle or bone marrow.^20,26^ We speculated that the differences in the distribution of lymphatic vessels was one of the reasons for the higher incidence of lymph node metastasis in cases with tumors involving the subcutis than in those with tumors involving muscles or bones.

Most previous reports showed that the frequency of lymph node metastasis in malignant soft tissue tumors was less than 5%.^10,11^ The rate of lymph node metastasis was relatively high (29/242 = 12.0%) in the current study, the probable reasons for which were as follows: our study excluded intermediate tumors (58 cases), included patients with lymph node metastasis pre-treatment (12 cases) and used FDG-PET for whole body screening. FDG-PET was previously reported to be useful for whole body screening of metastatic malignant bone and soft tissue tumors.^27^ We used FDG-PET to scan 20 of the 29 patients who had lymph node metastasis in this study, and diagnosed 19 as positive, meaning that this modality was also useful to detect lymph node metastases in patients with malignant bone and soft-tissue tumors. Modalities to improve the accuracy of the detection of metastasis in the whole body, such as FDG-PET, will likely lead to a higher rate of detection of lymph node metastases in patients with malignant bone and soft-tissue tumors than that have been reported in previous studies.

There are some limitations associated with this study. First, the purpose of this study was to identify the risk factors for lymph node metastasis, and therefore, patients with metastasis at the first visit and patients receiving only palliative treatment were included, which made it difficult to evaluate the effects of curative treatment, such as radiation, surgery, and chemotherapy, on metastasis. Second, the reason why lymphatic histotypes, including rhabdomyosarcoma, epithelioid sarcoma, and clear cell sarcoma, tended to metastasize to lymph nodes remains unknown. The lymphatic histotype was a risk factor for lymph node metastasis independent from other risk factors, including the histological location of the primary tumor and local recurrence, and therefore, the microenvironment of the tumors and tumor cell motility probably cannot explain this finding. Some previous reports described that VEGF-C and -D stimulated lymphangiogenesis via their receptors and were related to lymphatic metastasis.^15–17^ A previous in vitro study showed that VEGF-D enhanced the transendothelial migration of sarcoma cells through the human lymphatic endothelial monolayer more than VEGF-C.^18^ Analyzing the expression of genes related to lymphangiogenesis in cases with the lymphatic histotype may answer this question.

Lymph node metastasis was found in malignant bone and soft-tissue tumors more frequently than that was previously reported, and the risk factors for lymph node metastasis were the lymphogenous histotype and primary tumors invading the subcutis. The results of this study will contribute to the prevention of lymph node metastasis in patients with malignant bone and soft-tissue tumors, or may be useful to stratify patients to a particular treatment based on their risk of metastasis.
